# Risk indicators of oral health status among young adults aged 18 years analyzed by negative binomial regression

**DOI:** 10.1186/1472-6831-13-40

**Published:** 2013-08-19

**Authors:** Hai-Xia Lu, May Chun Mei Wong, Edward Chin Man Lo, Colman McGrath

**Affiliations:** 1 Department of Preventive Dentistry, Ninth People’s Hospital, Shanghai Jiao Tong University, School of Medicine, Shanghai Key Laboratory of Stomatology, Shanghai, China; 2 Dental Public Health, Faculty of Dentistry, University of Hong Kong, 34 Hospital Road, Hong Kong, China

**Keywords:** Dental caries, Periodontal disease, Negative binomial regression

## Abstract

**Background:**

Limited information on oral health status for young adults aged 18 year-olds is known, and no available data exists in Hong Kong. The aims of this study were to investigate the oral health status and its risk indicators among young adults in Hong Kong using negative binomial regression.

**Methods:**

A survey was conducted in a representative sample of Hong Kong young adults aged 18 years. Clinical examinations were taken to assess oral health status using DMFT index and Community Periodontal Index (CPI) according to WHO criteria. Negative binomial regressions for DMFT score and the number of sextants with healthy gums were performed to identify the risk indicators of oral health status.

**Results:**

A total of 324 young adults were examined. Prevalence of dental caries experience among the subjects was 59% and the overall mean DMFT score was 1.4. Most subjects (95%) had a score of 2 as their highest CPI score. Negative binomial regression analyses revealed that subjects who had a dental visit within 3 years had significantly higher DMFT scores (IRR = 1.68, p < 0.001). Subjects who brushed their teeth more frequently (IRR = 1.93, p < 0.001) and those with better dental knowledge (IRR = 1.09, p = 0.002) had significantly more sextants with healthy gums.

**Conclusions:**

Dental caries experience of the young adults aged 18 years in Hong Kong was not high but their periodontal condition was unsatisfactory. Their oral health status was related to their dental visit behavior, oral hygiene habit, and oral health knowledge.

## Background

The need for population-based oral epidemiological studies has long been advocated to determine the oral health needs of a population, set targets for the future, and to plan oral health services appropriately [1]. Prevalence and severity of dental caries experience in 12-year-old children in most industrialized countries is quite low, as well as in Hong Kong [2]. However, the dental caries situation among adults is generally unsatisfactory, as it tends to be in the worldwide population [3]. According to the Hong Kong Oral Health Survey conducted in 2001 [4], the prevalence of dental caries experience among 12-year-olds was 37.8% and their mean DMFT score of 0.8 suggested that the oral health condition of 12-year-olds could be regarded as very good in terms of the dental caries level. However, the prevalence of dental caries for the adults aged 35 to 44 years was 97.5% with a mean DMFT score of 7.4, the dental caries level being considerably higher. Due to the complete lack of oral health information on young adults, it is impossible to understand the dramatic changes in oral health status from childhood to adulthood. Therefore, it is of great importance to collect oral health information on young adults. Although some studies have been conducted among young adults such as 18 year-olds in other countries and areas, these studies mostly reported on dental caries, periodontal disease and malocclusion descriptively [5-9]. Studies with comprehensive profile of this population group with the intension to explore the risk indicators or factors of dental caries and periodontal disease are rare and no such comprehensive data is available in Hong Kong.

In oral epidemiology, the DMF/dmf index, a measure of cumulative caries experience of the teeth or surfaces, has been recommended by World Health Organization [1] and is widely used in dental research. This gives rise to count data that are commonly collected in medical and dental research. In epidemiological study, a count is defined as the number of occurrences of an event during a fixed time period, which is reported in non-negative values including zero and positive integers [10]. During the last three decades, dental caries has declined both for children and adults in many industrialized countries, as well as in Hong Kong [2]. For instance, the prevalence of dental caries experience in permanent dentition for 12-year-old age group had declined from 80% in 1968 to 37.8% in 2001 in Hong Kong [4,11]. Thus, the distribution of DMFT/dmft score has become increasingly skewed to the right and the proportion of zero scores (DMFT/dmft score = 0) has increased over time [12]. The standard assumption of normality cannot be held for such data, even with data transformation. However, such count data have erroneously been treated as ratio data, and multiple linear regressions using ordinary least square regression based on normal distribution have been inappropriately used for quite a long time [13]. The use of ordinary least square regression may be potentially biased leading to invalid findings [14], thus it is important to analyze such count data using appropriate statistical models.

When constructing regression models for DMFT/dmft score, the appropriate starting point is Poisson regression [13,15]. An important assumption of Poisson regression is equidispersion which indicates that the mean is equal to the variance of the distribution. However, this assumption is violated in most real life data where the variances appeared to be larger than the mean values [15]. Therefore, negative binomial regression was introduced to model the count data with overdispersion (that is, the variance is greater then the mean) [16,17]. Studies have indicated that the use of negative binomial regression is a more suitable modeling approach for dental caries data compared with Poisson regression due to the overdispersion of data [18-21]. In cases where the proportion of “zero” caries counts is in excess of what can be handled by the Poisson or negative binomial regressions, zero-inflated Poisson or negative binomial regressions should be considered [22].

When periodontal health status is recorded using Community Periodontal Index (CPI) in an epidemiological study, the highest CPI score for each individual is always reported. However, in some situations, the variability of the highest CPI score is small, that is, the majority of the subjects have the highest CPI score indicates presence of calculus (score 2) or shallow periodontal pockets (score 3). Use of the highest CPI score as the outcome variable to construct the regression model is not feasible due to a lack of variability of data. The number of sextants with healthy gums for each individual is an alternative measure to represent the periodontal health status of subjects. Again Poisson, negative binomial, zero-inflated Poisson or zero-inflated negative binomial regression can be used to model such count data depending on the extent of overdispersion.

The aim of this study was to investigate the oral health status (dental caries experience and periodontal health status) of young adults and to explore risk indicators of oral health status of young adults in Hong Kong analyzed by negative binomial or zero-inflated negative binomial regression models to handle the issue of overdispersion.

## Methods

### Sample

The study sample was Hong Kong young adults aged 18 years. This sample originally came from a population-based cohort study which started in 2001 when the subjects aged 12 years, and was followed up in 2007 when the subjects aged 18 years. In the 2001 Hong Kong Oral Health Survey conducted by the Hong Kong government [4], the sampling frame was the list of all local secondary schools in Hong Kong provided by the Education Department. A total of 26 schools were randomly selected using systematic sampling (every one in 23 schools was selected systematically from the list). Finally, 18 schools agreed to participate. All students in the survey were born in the year 1988 and were then 12 years old. Up to 50 students were randomly selected using systematic sampling from each of the selected schools owing to the limited resources and avoidance of inordinate disturbance of classes. Finally, 638 children participated in 2001 survey. In 2007, when the children reached the age of 18 years, attempts to contact all of them were made. The data described here derived from follow-up study in 2007.

Ethical approval for this study was obtained from the University of Hong Kong Institutional Review Board prior to the implementation of the study, and written informed consent was obtained from the participants or their parent as appropriate.

### Data collection

The data collection consisted of two parts: a clinical examination and a questionnaire survey. Subjects were clinically examined in the 2007 follow-up and information on their oral health status including dental caries experience and periodontal health status was collected. Dental caries experience of the subjects was recorded by counting the number of teeth that were decayed (D), missing due to caries (M), and filled (F) for calculation of the DMFT score according to criteria propose by World Health Organization [1]. Dental caries was detected visually at the cavitation level and early caries was not recorded. Periodontal health status was assessed by the Community Periodontal Index (CPI) according to WHO criteria [1]. The CPI score was recorded as following: 0- Healthy; 1- Bleeding after probing; 2- Calculus; 3- Pocket 4–5 mm; 4- Pocket 6 mm or more. The highest CPI score for each sextant was recorded from six index teeth (they were 16, 11, 26, 36, 31 and 46). The number of sextants with healthy gums of each subject (CPI = 0) was generated as the main outcome variable for periodontal health status.

The examinations were conducted in the schools if subjects were still studying in the school. For those subjects who had left school to work or studied abroad, the examinations took place in the Prince Philip Dental Hospital. The subjects were examined by a trained and calibrated examiner using a disposable mouth-mirror attached to an intraoral LED light and lightweight CPI probes. To monitor the intra-examiner reproducibility, 10% of subjects were re-examined. Intra-examiner reliability on caries status at tooth level and periodontal conditions, as measured by Kappa statistics, was 0.99 and 0.89, respectively.

After the clinical examination, subjects were asked to self-complete a structured questionnaire. Information on their oral health related behaviors (such as snacking frequency, toothbrushing frequency, use of fluoride toothpaste, dental floss, toothpick, mouthrinse, and utilization of dental services during past three years), oral health knowledge, and oral health attitudes were collected in the questionnaire.

Two scores to measure the oral health knowledge and attitudes of the subjects were adopted in this study [23,24]. In order to measure the oral health knowledge, four questions regarding the causes and the prevention of dental caries and gum disease were asked and no more than three acceptable answers were recorded for each question. An oral health knowledge score was computed by counting the total number of acceptable answers given by the subjects. However, answers like “do not know” and “no answer” were excluded. Thus, the oral health knowledge score was an interval variable with a range of 0 to 12. A higher oral health knowledge score indicates better oral health knowledge. Regarding the oral health attitudes score, eight statements regarding the importance of oral health, the importance of retaining natural teeth, utilization of dental service, and oral health beliefs were adopted to evaluate the oral health attitude. The subjects were asked to indicate whether they agree with, disagree with, or had no comment on each statement. Similar to the oral health knowledge score, oral health attitudes score was computed by counting the total number of statements to which the subjects held a positive attitude. Similar to the oral health knowledge score, the oral health attitudes score was an interval variable with a range of 0–8. A higher attitudes score indicates more positive attitudes.

Parents or guardians of subjects were asked by telephone to answer the questions on the family socio-demographic characteristics of the subjects (parental educational attainment and monthly household income level). All questionnaires were printed in Chinese.

### Data analysis

Descriptive statistics (means, standard deviation and percentage) of the family socio-demographic characteristics, oral health related behaviors, knowledge, and attitudes were presented. In order to investigate the associations between family socio-demographic background, oral health related behaviors, knowledge, attitudes and the clinical oral health status among 18-year-old young adults, bivariate analyses were performed. First of all, Chi square tests were performed to assess the difference in the prevalence of dental caries experience among different groups. Secondly, Mann–Whitney U tests or Kruskal Wallis one-way ANOVA tests to compare the distribution of DMFT scores and the distribution of number of sextants with healthy gums among different groups as the data were not normally distributed. Finally, Spearman’s rank correlation coefficients were used to assess the association between DMFT score, number of sextants with healthy gums and oral health knowledge and attitudes score. The data were analyzed using SPSS 16.0 (SPSS Inc, Chicago, IL, USA). The statistical significance level for all tests was set to be 0.05.

To explore the risk indicators of dental caries experience and periodontal health status, negative binomial and zero-inflated negative binomial regressions were both considered and compared by the Vuong test. Negative binomial regression would be chosen if the p-value of the Vuong test was not significant (p > 0.05) indicating the zero-inflated negative binomial regression was not significantly better than the negative binomial regression. All four socio-demographic and nine other covariates were included in the regression models. Insignificant covariates while adjusting for the socio-demographic factors were removed by backward elimination until only covariates that showed significant association (p < 0.05) were retained. In order to adjust for the possible clustering effect that some young adults were from the same school, incidence rate ratio (IRR) for each indicator was estimated with the use of the Huber-White sandwich estimate for standard errors (robust estimates). Model fit was assessed, for a good model fit, the likelihood ratio test should have p-value > 0.05 when comparing to the full model containing all the covariates suggesting the fitted model was not statistically different from the full model. The above analyses were performed using STATA version 12 (StataCorp, College Station, Texas, USA).

## Results

### Oral health status

**Figure 1:**
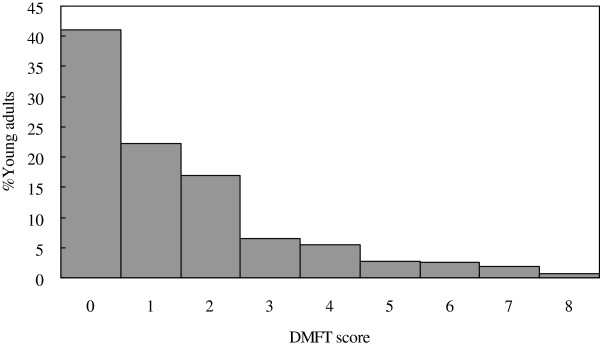
Frequency distribution of the studied young adults according to DMFT score (n = 324).

**Figure 2:**
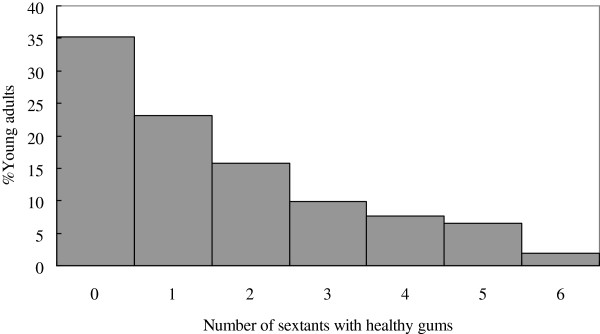
Frequency distribution of the studied young adults according to the number of sextants with healthy gums (n = 324).

A total of 324 young adults aged 18 years (male: 177 and female: 147) were examined in the 2007 follow-up. The overall mean DMFT score was 1.4 (DT: 0.3, MT: <0.1, and FT: 1.1) with filled teeth accounting for around 80% of the dental caries experience. The frequency distribution of DMFT score was highly skewed with excessive zero score (Figure 1). Prevalence of caries experience among the subjects was 59.0%. Only 7.8% of the subjects had 5 or more teeth with dental caries experience. Most of subjects (93.5%) had a score of 2 (calculus) as their highest CPI score, while only 1.9% had healthy gums (CPI = 0). The mean number of sextants with healthy gums was 1.59 while mean number of sextants with bleeding (CPI = 1), calculus (CPI = 2), and shallow pockets (CPI = 3) were 0.61, 3.76, and 0.04, respectively. As shown in Figure 2, the frequency distribution of the number of sextants with healthy gums of the studied young adults was highly skewed as well.

### Oral health related behaviors, knowledge and attitudes

Almost one third of the subjects (32.4%) reported to snack twice or more daily and females tended to snack more frequently than males (42.2% *vs*. 24.3%, p = 0.001). More than two thirds of them (67.9%) brushed their teeth twice or more daily and generally females reported to brush their teeth more frequently than males (81.6% *vs*. 56.5%, p < 0.001). Over half of them (55.6%) reported to use fluoride toothpaste, and no significant difference among males and females was found (p > 0.05). The majority of the subjects reported not to use additional oral cleaning aids, specifically dental floss (nearly 80% did not use the dental floss). Again, more females used dental floss and toothpick when compared to males (30% *vs.* 13%, p < 0.001 and 43% *vs.* 28%, p = 0.006, respectively). Among these 324 subjects, nearly half of them had utilized dental services during last three years (49.4%). However, fewer than 20% of the subjects had regular dental visits.

Regarding oral health knowledge, all subjects had high knowledge scores with a mean score of 10.5 (SD = 2.3). A large proportion of the subjects held positive attitudes to oral health. The oral health attitudes score was calculated for each subject and the mean score was 6.3 (SD = 1.3).

### Risk indicators associated with oral health status

The associations between dental caries experience (including prevalence of caries experience and mean DMFT score), mean number of sextants with healthy gums of the studied young adults and their socio-demographic background are shown in Tables 1 and 2. Among these variables, only a significant gender difference in the distribution of the number of sextants with healthy gums score was found, with females had significantly higher number of sextants with healthy gums than males (p = 0.001).

**Table 1:** Dental caries experience of the studied young adults according to their socio-demographic background (n = 324)

	**n**	**% with DMFT > 0**	p^*^	**Mean DMFT (SD)**	p^**^
Gender			0.595		0.311
Male	177	57.6		1.4 (1.7)	
Female	147	60.5		1.6 (1.8)	
Parental educational attainment			0.265		0.222
Primary or below	39	64.1		1.9 (2.3)	
Junior high school	82	67.1		1.7 (1.8)	
Senior high school	134	53.7		1.3 (1.6)	
Matriculation or above	32	62.5		1.3 (1.4)	
Do not know	37	51.4		1.3 (1.9)	
Monthly household income level			0.188		0.095
Below HK$20,000	236	61.0		1.6 (1.9)	
Above HK$20,000	78	52.6		1.1 (1.4)	
Dental insurance coverage			0.826		0.871
No	218	59.6		1.5 (1.7)	
Yes	31	61.3		1.2 (1.3)	
Do not know	75	56.0		1.6 (2.1)	

^*^ p-value was computed from Chi square test to compare the proportions between groups.

^**^ p-value was computed from Mann–Whitney *U* test or Kruskal Wallis 1-way ANOVA test to compare the distributions between groups.

**Table 2:** The mean number of sextants with healthy gums of the studied young adults according to their socio-demographic background (n = 324)

	**n**	**Mean Number of sextants with healthy gums (SD)**	p^*^
Gender			0.001
Male	177	1.38 (1.71)	
Female	147	1.83 (1.58)	
Parental educational attainment			0.797
Primary or below	39	1.69 (1.81)	
Junior high school	82	1.46 (1.65)	
Senior high school	134	1.57 (1.65)	
Matriculation or above	32	1.78 1.54)	
Do not know	37	1.65 1.75)	
Monthly household income level			0.593
Below HK$20,000	236	1.57 (1.67)	
Above HK$20,000	78	1.62 (1.58)	
Dental insurance coverage			0.948
No	218	1.58 (1.62)	
Yes	31	1.65 (1.80)	
Do not know	75	1.59 (1.76)	

^*^ p-value was computed from Mann–Whitney *U* test or Kruskal Wallis 1-way ANOVA test to compare the distributions between groups.

Table 3 presents the relationships between dental caries experience of the studied young adults and their oral health related behaviors. It was also found that subjects who had utilized dental services had higher DMFT and FT scores than those who did not (p = 0.004 and p < 0.001 respectively). No other significant relationships could be found with other behaviors such as snacking frequency, toothbrushing frequency, use of fluoride toothpaste and use of dental floss (p > 0.05). Dental caries experience of subjects was also found to be not significantly associated with oral health knowledge score and attitudes score (r_s_ = 0.007, p = 0.903 and r_s_ = 0.044, p = 0.427, respectively).

**Table 3:** Dental caries experience of the studied young adults according to their behaviors (n = 324)

	**n**	**% with DMFT > 0**	p^*^	**Mean DMFT (SD)**	p^**^
Snacking frequency			0.355		0.500
Never	88	62.5		1.3 (1.4)	
Once	131	61.1		1.7 (2.1)	
Twice or more	105	53.3		1.3 (1.7)	
Toothbrushing frequency			0.867		0.280
Once or less	104	59.6		1.1 (1.3)	
Twice or more	220	58.6		1.6 (1.9)	
Use of fluoride toothpaste			0.840		0.731
With fluoride	180	59.4		1.4 (1.7)	
No fluoride or do not know	144	58.3		1.5 (1.9)	
Use of dental floss			0.675		0.456
No	257	58.4		1.4 (1.8)	
Yes	67	61.2		1.5 (1.8)	
Use of mouthrinse			0.107		0.091
No	238	56.3		1.4 (1.9)	
Yes	86	66.3		1.6 (1.5)	
Use of toothpick			0.702		0.911
No	211	59.7		1.4 (1.8)	
Yes	113	57.5		1.5 (1.8)	
Utilization of dental services during past 3 years			0.050		0.004
No	164	53.7		1.1 (1.6)	
Yes	160	64.4		1.8 (1.9)	

^*^ p-value was computed from Chi square test to compare the proportions between groups.

^**^ p-value was computed from Mann–Whitney *U* test or Kruskal Wallis 1-way ANOVA test to compare the distributions between groups.

Regarding the relationships between the mean number of sextants with healthy gums and their oral health related behaviors (Table 4), subjects who brushed their teeth twice or more daily had significantly higher number of sextants with healthy gums than those who only brushed their teeth once daily or less frequently (p < 0.001). A significantly higher number of sextants with healthy gums was found in the subjects who used dental floss (p = 0.036). Utilization of dental services during the past three years also had a significant positive association with the number of sextants with healthy gums (p = 0.001). Furthermore, oral health knowledge and attitudes scores were positively correlated with the number of sextants with healthy gums (r_s_ = 0.129, p = 0.021 and r_s_ = 0.149, p = 0.007, respectively), with subjects who had better oral health knowledge and held more positive attitudes toward oral health having better periodontal status.

**Table 4:** Periodontal status of the studied young adults according to their behaviors (n = 324)

	**Number of sextants with healthy gums**		
	**n**	**Mean (SD)**	p^*^
Snacking frequency			0.446
Never	88	1.4 (1.6)	
Once	131	1.6 (1.8)	
Twice or more	105	1.7 (1.6)	
Toothbrushing frequency			<0.001
Once or less	104	0.9 (1.3)	
Twice or more	220	1.9 (1.7)	
Use of fluoride toothpaste			0.022
With fluoride	180	1.8 (1.7)	
No fluoride or do not know	144	1.4 (1.6)	
Use of dental floss			0.036
No	257	1.5 (1.7)	
Yes	67	1.9 (1.7)	
Use of mouthrinse			
No	238	1.6 (1.6)	0.812
Yes	86	1.7 (1.7)	
Use of toothpick			
No	211	1.6 (1.6)	0.620
Yes	113	1.7 (1.7)	
Utilization of dental services during past 3 years			0.001
No	164	1.3 (1.6)	
Yes	160	1.9 (1.7)	

^*^ p-value was computed from Mann–Whitney *U* test or Kruskal Wallis 1-way ANOVA test to compare the distributions between groups.

### Negative binomial regressions

In order to identify the effects of the above factors on the DMFT scores, all the above mentioned covariates were considered in the negative binomial and zero-inflated regression models. By Vuong test, zero-inflated negative binomial regression model was not significantly better than negative binomial model (z = 1.14, p = 0.128), thus negative binomial model was chosen. In the final regression model adjusted for the socio-demographic factors (Table 5), it was found that the mean DMFT score of subjects who utilized dental services within the last 3 years was 1.68 times (i.e. 68% higher) compared to that of subjects who did not utilize (IRR = 1.68, p < 0.001). The model fitted the data well with p-value of likelihood ratio test = 0.573.

**Table 5:** Relationships between DMFT score of the studied young adults and selected independent variables (Negative binomial regression) (n = 324)

	**Incidence rate ratio**	**95% CI**^**#**^	**p**
Utilization of dental services during past three years			
Yes	1.68	1.29-2.18	<0.001
No ^a^			

The model was adjusted by all four the socio-demographic factors: Gender, education level, income household income and dental insurance coverage.

^a^ Reference group. ^#^95% CI = 95% Confidence Interval.

For the number of sextants with healthy gums, negative binomial regression was chosen as well (Vuong test: z = 0.65, p = 0.257). In the final regression model adjusted for the socio-demographic factors, two factors (toothbrushing frequency and oral health knowledge score) showed significant associations with the mean number of sextants with healthy gums (Table 6). Subjects who brushed their teeth more frequently (IRR = 1.93, p < 0.001) and those with better oral health knowledge (IRR = 1.09, p = 0.002) had significantly more sextants with healthy gums. The negative binomial regression showed a good fit with p-value of likelihood ratio test = 0.687.

**Table 6:** Relationship between mean numbers of sextants with healthy gums of the studied young adults and selected independent variables (Negative binomial regression) (n = 324)

	**Incidence rate ratio**	**95% CI**^**#**^	**p**
Toothbrushing frequency			
Twice or more	1.93	1.43-2.60	<0.001
Once or less ^a^			
Oral health knowledge score	1.09	1.03-1.15	0.002

The model was adjusted by all four the socio-demographic factors: Gender, education level, income household income and dental insurance coverage.

^a^ Reference group. ^#^95% CI = 95% Confidence Interval.

## Discussion

In this study, the study sample was not selected directly using a random sampling method for the 18 years old young adults. It would be difficult if not impossible to construct an appropriate sampling frame for them as some of them may be studying in the last year of the secondary education, some of them may have been studying in the tertiary institutions and some others may have been working. It was actually derived from following up a random and representative sample of 12-year-old children who participated in the Hong Kong Oral Health Survey conducted by the government in 2001. Although the follow-up rate was not high (50.8%), a comparison of the socio-demographic background and baseline oral health status (DMFT score and highest CPI score) of the subjects who were followed up and those who were lost to follow-up was made and no statistically significant differences was found between these two groups [25]. Thus, the representativeness of the sample in this study to represent the population of Hong Kong young adults aged 18 years could be maintained.

In the present study, negative binomial and zero-inflated negative binomial regression models, as a member of the Generalized Linear Models (GZLM) [17,22], were performed to illustrate the appropriate way to analyze non-negative integer count data such as DMFT scores and the number of sextants with healthy gums. GZLM are the extension of the linear regression which can be used for modeling different types of outcome variables such as binary, ordered categorical, count and survival data [10,17]. It can fit the data that follow various probability distributions other than the normal distribution, such as the Poisson, Binomial, multinomial and so on. In the current study, negative binomial regression models were found to fit the data well enough and the use of zero-inflated models was unnecessary. However, it is important to compare the alternative models using statistical test and choose the appropriate models accordingly. Furthermore, it is noteworthy that the covariate effects in Poisson and negative binomial regression models are multiplicative whereas covariate effects in multiple linear regression model are additive.

With the introduction of water fluoridation since 1960s in Hong Kong, the dental caries prevalence and experience had been declining dramatically in the early years, but had stabilized from the 1980s onwards. Thus, it is not surprising to observe that the prevalence and experience of dental caries of young adults in this study were at a similar level as those of the 15–19 year-olds in 1984 (prevalence: 59% *vs.* 58%; mean DMFT: 1.4 *vs.* 1.7, respectively) [26]. However, there was still a change in the main component of DMFT score from DT in 1984 to FT in this study due to the introduction of School Dental Care Services (SDCS). The SDCS for primary school children was established in 1979 in Hong Kong and some basic oral health care including dental examination, filling, scaling, simple extraction and oral health education is provided by government without any charge [27].

A comparison of the dental caries experience of young adults aged 18 years in this study and those in other countries was made. Dental caries situations of young adults in Hong Kong were much better compared to some developing countries such as Iran and Burkina Faso [7,9]. Even when compared with the developed countries [8,28-30], the dental caries experience of the 18 year-olds in Hong Kong young adults is found to be lower. Although the mean DMFT scores of the young adults in Hong Kong and Mainland China young adults are similar, the components are completely different [31]. Due to SDCS, a large proportion of DMFT score in Hong Kong young adults was contributed by filled teeth, while that in Mainland China was dominated by decayed teeth. The same pattern of components was also found in other countries, with decayed teeth and filled teeth dominating the DMFT score of young adults in the developing countries and the developed countries, respectively.

Although a number of indicators including oral health related behaviors, knowledge and attitudes were considered in the negative binomial regression model on dental caries experience, only utilization of dental service during past three years were identified to be associated with the dental caries experience of the subjects. This finding is in agreement with review paper regarding risk factors of dental caries in some extent [32]. Problem-oriented dental visiting pattern were found among these young adults. The majority of subjects in this study visited a dentist only when they had a problem that required treatment and not for preventive care. It was found that young adults who had a greater utilization of dental services had higher dental caries experience and a higher FT component. Due to the predominant item-based fee-for-service payment arrangement in the dental market in Hong Kong, dentists typically adopt a curative or restorative rather than a preventive approach when treating patients. Although this finding was obtained from a cross-sectional study rather than longitudinal study in nature, the present finding is consistent with results of a cohort study which followed up this population from 12 to 18 years of age [25], in which childhood economic circumstance of the subjects was found to be positively related to their utilization of dental services and then utilization of dental services contributed to the subjects’ dental caries experience. This further confirmed that family economic circumstance and utilization of dental services were the important factors which did affect the dental caries level in young adults.

Due to very few studies reporting the periodontal status using the highest CPI score of 18 years old age group, the comparisons are only made with Iran and Mainland China. Comparing to Iranian young adults [7], the periodontal status in Hong Kong young adults can be regarded as better, with proportionally fewer young adults having shallow pockets. However, it seems that the periodontal status of the young adults in Hong Kong is worse than Mainland China [31], with proportionally more young adults having calculus.

In the final negative binomial regression model for periodontal health status, only toothbrushing frequency and oral health knowledge score exerted significant effects on the number of sextants with healthy gums. This finding is consistent with the results of cohort study which followed up this population from 12 to 18 years of age [25], in which both socioeconomic background and utilization of dental services had no significant effects on the periodontal health status, which indicated that some other factors may affect a subjects’ periodontal health status. Unfortunately, oral health behaviors data of this cohort was not available when these young adults were aged 12 or 15 years. The results found from the cross-sectional data provided valuable information on the factors associated with periodontal health status.

It was noteworthy that the studied young adults with better oral health related knowledge had more favorable periodontal status. Young adults who knew more about the causes and prevention methods of periodontal disease would avoid those undesirable behaviors and practice more proper oral health behaviors in daily life. It was also found that the studied young adults who brushed their teeth more frequently had more favorable periodontal status. Since dental plaque plays an important role in the development of gingival inflammation and chronic periodontitis [33], it is logical that mechanical removal of the dental plaque helps maintaining good health of the periodontal tissue. More oral health promotion should target on this aspect.

## Conclusions

The prevalence of caries experience of the young adults in Hong Kong was not high but their periodontal condition was unsatisfactory. Their oral health status was related to their dental visit behavior, oral hygiene habit, and oral health knowledge. The negative binomial regression model was appropriate in analyzing non-negative integers count data such as DMFT score and the number of periodontally healthy sextants.

## Competing interest

The authors declare that there has no competing interest.

## Authors’ contributions

HXL participated in the conception and design of the study, data acquisition, data analysis and drafting the manuscript. MCMW contributed to the conception and design of the study, data analysis, interpretation and critical revision of manuscript. ECML participated in the conception and design of the study, data collection and critical revision of manuscript. CM contributed to the conception and design of the study and critical revision of manuscript. All authors read and approved the final manuscript.

## Pre-publication history

The pre-publication history for this paper can be accessed here:

http://www.biomedcentral.com/1472-6831/13/40/prepub
